# Nucleocapsid (N) Gene Mutations of SARS-CoV-2 Can Affect Real-Time RT-PCR Diagnostic and Impact False-Negative Results

**DOI:** 10.3390/v13122474

**Published:** 2021-12-10

**Authors:** Jéssika Cristina Chagas Lesbon, Mirele Daiana Poleti, Elisângela Chicaroni de Mattos Oliveira, José Salvatore Leister Patané, Luan Gaspar Clemente, Vincent Louis Viala, Gabriela Ribeiro, Marta Giovanetti, Luiz Carlos Junior de Alcantara, Olivia Teixeira, Maria Cristina Nonato, Loyze Paola Oliveira de Lima, Antonio Jorge Martins, Claudia Renata dos Santos Barros, Elaine Cristina Marqueze, Jardelina de Souza Todão Bernardino, Debora Botequio Moretti, Ricardo Augusto Brassaloti, Raquel de Lello Rocha Campos Cassano, Pilar Drummond Sampaio Correa Mariani, Svetoslav Nanev Slavov, Rafael Bezerra dos Santos, Evandra Strazza Rodrigues, Elaine Vieira Santos, Josiane Serrano Borges, Debora Glenda Lima de La Roque, Joao Paulo Kitajima, Bibiana Santos, Patricia Akemi Assato, Felipe Allan da Silva da Costa, Cecilia Artico Banho, Livia Sacchetto, Marilia Mazzi Moraes, Melissa Palmieri, Fabiana Erica Vilanova da Silva, Rejane Maria Tommasini Grotto, Jayme A. Souza-Neto, Mauricio Lacerda Nogueira, Luiz Lehman Coutinho, Rodrigo Tocantins Calado, Raul Machado Neto, Dimas Tadeu Covas, Simone Kashima, Maria Carolina Elias, Sandra Coccuzzo Sampaio, Heidge Fukumasu

**Affiliations:** 1 Laboratory of Comparative and Translational Oncology, Department of Veterinary Medicine, School of Animal Science and Food Engineering, University of Sao Paulo, Pirassununga 13635-900, São Paulo, Brazil; jessika.chagas@usp.br (J.C.C.L.); mirelep@usp.br (M.D.P.); limattos@usp.br (E.C.d.M.O.); 2 Butantan Institute, São Paulo 05503-000, São Paulo, Brazil; jose.patane@butantan.gov.br (J.S.L.P.); vincent.viala@butantan.gov.br (V.L.V.); gabriela.ribeiro.br@gmail.com (G.R.); loyze.lima@butantan.gov.br (L.P.O.d.L.); antonio.martins@butantan.gov.br (A.J.M.); claudia.barros@butantan.gov.br (C.R.d.S.B.); elaine.marqueze@butantan.gov.br (E.C.M.); jardelina.bernardino@butantan.gov.br (J.d.S.T.B.); debora.moretti@butantan.gov.br (D.B.M.); raul.machado@butantan.gov.br (R.M.N.); dimas.covas@butantan.gov.br (D.T.C.); carolina.eliassabbaga@butantan.gov.br (M.C.E.); sandra.coccuzzo@butantan.gov.br (S.C.S.); 3 Functional Genomic Center, Department of Animal Science, Luiz de Queiroz School of Agriculture, University of Sao Paulo, Piracicaba 13418-900, São Paulo, Brazil; luan.clemente@usp.br (L.G.C.); ricardo.brassaloti@usp.br (R.A.B.); raquel_lello@yahoo.com.br (R.d.L.R.C.C.); llcoutinho@usp.br (L.L.C.); 4 Fundação Oswaldo Cruz, FIOCRUZ, Manguinhos 21040-900, Rio de Janeiro, Brazil; marta.giovanetti@ioc.fiocruz.br (M.G.); alcantaraluiz42@gmail.com (L.C.J.d.A.); 5 Institute of Biological Sciences, Federal University of Minas Gerais, Belo Horizonte 31270-901, Minas Gerais, Brazil; 6 Ribeirao Preto Protein Crystallography Laboratory, School of Pharmaceutical Sciences, University of São Paulo, Ribeirao Preto 14040-903, São Paulo, Brazil; olivia.teixeira@usp.br (O.T.); cristy@fcfrp.usp.br (M.C.N.); 7 NGS Soluções Genômicas, Piracicaba 13416-030, São Paulo, Brazil; pilarmariani@hotmail.com; 8 Blood Center of Ribeirão Preto, Ribeirão Preto Medical School, University of São Paulo, Ribeirão Preto 14051-060, São Paulo, Brazil; svetoslav.slavov@hemocentro.fmrp.usp.br (S.N.S.); rafaelbezerra@usp.br (R.B.d.S.); evandra@hemocentro.fmrp.usp.br (E.S.R.); elainevs@alumni.usp.br (E.V.S.); josiane.boges@hemocentro.fmrp.usp.br (J.S.B.); debora.laroque@gmail.com (D.G.L.d.L.R.); rtcalado@usp.br (R.T.C.); skashima@hemocentro.fmrp.usp.br (S.K.); 9 Mendelics Genomic Analysis, São Paulo 02511-000, São Paulo, Brazil; joao.kitajima@mendelics.com.br (J.P.K.); bibiana.santos@mendelics.com.br (B.S.); 10 School of Agricultural Sciences, São Paulo State University, Botucatu 18618-970, São Paulo, Brazil; p.assato@unesp.br (P.A.A.); felipe.allan@unesp.br (F.A.d.S.d.C.); rejane.grotto@unesp.br (R.M.T.G.); jayme.souza-neto@unesp.br (J.A.S.-N.); 11 Laboratório de Pesquisas em Virologia, Departamento de Doenças Dermatológicas, Infecciosas e Parasitárias, Faculdade de Medicina de São José do Rio Preto, São José do Rio Preto 15090-000, São Paulo, Brazil; ceci.abanho@gmail.com (C.A.B.); liviasacchetto@gmail.com (L.S.); mariliamazzi@hotmail.com (M.M.M.); mauricio.nogueira@edu.famerp.br (M.L.N.); 12 Coordenação de Vigilância em Saúde—Secretaria Municipal da Saúde, São Paulo 01223-906, São Paulo, Brazil; melissapalmieri@prefeitura.sp.gov.br; 13 Laboratory Assistance, Coordination of Primary Care, Municipal Health Department, São Paulo 01223-906, São Paulo, Brazil; fevilanova@prefeitura.sp.gov.br

**Keywords:** COVID-19, RT-PCR, *N* gene, GeneFinder, mutation

## Abstract

The current COVID-19 pandemic demands massive testing by Real-time RT-PCR (Reverse Transcription Polymerase Chain Reaction), which is considered the gold standard diagnostic test for the detection of the SARS-CoV-2 virus. However, the virus continues to evolve with mutations that lead to phenotypic alterations as higher transmissibility, pathogenicity or vaccine evasion. Another big issue are mutations in the annealing sites of primers and probes of RT-PCR diagnostic kits leading to false-negative results. Therefore, here we identify mutations in the *N* (Nucleocapsid) gene that affects the use of the GeneFinder COVID-19 Plus RealAmp Kit. We sequenced SARS-CoV-2 genomes from 17 positive samples with no *N* gene detection but with *RDRP* (RNA-dependent RNA polymerase) and E (Envelope) genes detection, and observed a set of three different mutations affecting the *N* detection: a deletion of 18 nucleotides (Del28877-28894), a substitution of GGG to AAC (28881-28883) and a frameshift mutation caused by deletion (Del28877-28878). The last one cause a deletion of six AAs (amino acids) located in the central intrinsic disorder region at protein level. We also found this mutation in 99 of the 14,346 sequenced samples by the Sao Paulo state Network for Pandemic Alert of Emerging SARS-CoV-2 variants, demonstrating the circulation of the mutation in Sao Paulo, Brazil. Continuous monitoring and characterization of mutations affecting the annealing sites of primers and probes by genomic surveillance programs are necessary to maintain the effectiveness of the diagnosis of COVID-19.

## 1. Introduction

The SARS-CoV-2 virus is the causative agent of the COVID-19 pandemic and real-time RT-PCR (Reverse Transcription Polymerase Chain Reaction) is the gold standard of diagnostic tests. However, there are several commercial and non-commercial tests available with different sensitivities and specificities, the majority of which were designed at the beginning of the pandemic with the available genome sequences at that moment. Twenty months after the first diagnosis, the virus evolved worldwide with different strains emerging on a daily basis. Therefore, mutations conferring phenotypic alterations as higher transmissibility, pathogenicity, or vaccine evasion happened as the ones described in the P.1 (Brazilian, Gamma), B.1.617 (Indian, Delta), and others. Mutations with unknown phenotypic consequences can occur on primers and probes annealing sites from the RT-PCR diagnostic kits. These mutations can lead to false-negative results, increasing the chance of false-negative COVID-19 patients spreading the virus.

To overcome this problem, most of the real-time RT-PCR COVID-19 diagnostic tests detect two or three SARS-CoV-2 gene fragments from *N* (Nucleocapsid), *E* (Envelope), *RdRP* (RNA-dependent RNA polymerase), *S* (spike), *ORF1* (Nonstructural protein), and others. However, there are protocols using only one SARS-CoV-2 gene fragment as is the case of recently developed tests for COVID-19 and Flu detection at the same time by multiplex real-time RT-PCR, increasing the possibility of false-negative SARS-CoV-2 virus detection.

At the beginning of March 2020, we set the Laboratory of Translational and Comparative Oncology of the School of Animal Science and Food Engineering of the University of Sao Paulo at Pirassununga, São Paulo State, Brazil, to fully work on COVID-19 diagnosis by real-time RT-PCR. Since then, we performed more than 130,000 tests for more than 40 cities from southeast of Sao Paulo State so far using three different protocols: the CDC (Centers for Disease Control and Prevention) (*N1*, *N2*, *RNP* Ribonucleoprotein), the GeneFinder COVID-19 Plus RealAmp Kit (*N*, *E*, *RdRP* and human control), and AllPlex 2019-nCoV Assay (*N*, *E*, *RdRP* and human control). Specifically, the GeneFinder kit was used on more than 90,000 samples from the Sao Paulo State COVID-19 Diagnostic Network which our laboratory also integrates. However, in early February 2021, we detected samples with the GeneFinder kit that had high viral load detected by *E* and *RdRP* probes but lacking *N* probe detection. This is a real problem since the *N* probe of the GeneFinder kit is the most sensitive for SARS-CoV-2 detection in our experience and samples with low viral load (Ct (Cycle threshold) > 33, i.e.,) are detected mainly by *N* probe detection.

Therefore, the objective of this work was to identify the possible mutation(s) in the SARS-CoV-2 *N* gene responsible for this specific problem and check if the problem was extended to other labs from the Sao Paulo State COVID-19 Diagnostic Network. Further characterization of the most frequent mutation in *N* gene affecting the diagnostics is also presented and discussed.

## 2. Materials and Methods

### 2.1. Diagnostic Service

The Laboratory of Translational and Comparative Oncology (LOCT-USP) from the School of Animal Science and Food Engineering (FZEA-USP, Pirassununga, SP, Brazil) is BSL-2 certified by CTNBio (Brazilian Technical Committee of Biosafety, Brasilia, DF, Brazil), certified by Instituto Adolfo Lutz for COVID-19 diagnostics and member of the Sao Paulo State COVID-19 Diagnostic Network and the Sao Paulo state Network for Pandemic Alert of Emerging SARS-CoV-2 variants.

### 2.2. Samples and Extraction of Total Nucleic Acids

Samples from nasopharyngeal and oropharyngeal swabs were collected according to the standard protocol and placed inside transport tubes containing 3 mL sterile saline solution. All samples were transported to the laboratory at a cold temperature (2–8 °C) within 12 h post collection and processed on the day. RNA was isolated from clinical samples (naso and oropharyngeal swabs) using the kit extract–RNA e DNA Viral (Loccus, Cotia, SP, Brazil). Purification by magnetic beads facilitates the isolation process and results in a high yield and purity level of the isolated nucleic acid.

### 2.3. Real-Time RT-PCR Using GeneFinder COVID-19 Plus RealAmp Kit

The majority of the real-time RT-PCR COVID-19 diagnostic tests were performed with the GeneFinder™ COVID-19 Plus RealAmp RT-PCR master mix (Osang Healthcare Co., Anyang-si, Korea). The purified nucleic acid is reverse transcribed into cDNA and can detect new coronavirus using a real-time RT-PCR probe, through specific primer using three different primers sets for viral detection (*N*, *E*, and *RdRP* gene fragments) and fluorescent probe reactions.

### 2.4. Genome Sequencing

To find out the *N* gene mutations, we selected 17 representative samples positive for COVID-19 by real-time RT-PCR, which did not present Ct values for the *N* gene. The libraries were constructed using Illumina COVIDSeq^TM^ Test (Illumina Inc, San Diego, CA, USA), according to manufacturer’s instructions. The cDNA was carried out on RNA samples isolated and synthesized by reverse transcriptase with random hexamers. The virus genome was amplified using two pool primers in separate PCR reactions. The PCR amplified product was processed for tagmentation and adapter ligation using IDT for Illumina Nextera UD Indexes Set A, B, C, D (384 indexes, 384 samples). The enrichment and cleanup steps were carried out according to the manufacturer’s protocol. All samples were processed as batches in a 96-well plate; these 96 libraries were pooled together in a tube. Pooled samples were quantified using Qubit dsDNA High Sensitivity assay kit on a Qubit fluorometer (Invitrogen Inc, Carlsbad, CA, USA), and the fragment sizes were analyzed in Agilent Fragment analyzer 5200 (Agilent Inc, Santa Clara, CA, USA). The pooled library was normalized to 4 nM concentration and denatured with 5 μL of 0.2 N of NaOH. The 1.2 pM library was spiked with 1% PhiX control (PhiX Control v3, Illumina Inc, San Diego, CA, USA) and sequenced on an Illumina MiniSeq plataform (Illumina), using a MiniSeq System Mid-Output Kit (300 cycles). The viral isolate sequences were aligned with the reference sequence for SARS-CoV-2 using the Illumina DRAGEN COVIDSeq Test pipeline. Viral strains were classified and mutations analyzed using the software tools Pangolin (http://pangolin.cog-uk.io/, accessed on 5 August 2021), and nextclade (https://clades.nextstrain.org/, accessed on 5 August 2021).

### 2.5. In Silico Evaluation of N Gene Mutations

To evaluate the possible structural impacts of the 203–208 deletion, and considering the fact that the structure of the full length protein is not known, we submitted the N protein sequence to the Robetta server (https://robetta.bakerlab.org, accessed on 10 August 2021) for structure prediction and analysis for both full length and mutated proteins. The default parameters were used to produce predicted models using the simultaneous processing of sequence, distance, and coordinate information by the three-track architecture implemented in the RoseTTAfold method [1].

## 3. Results

### 3.1. Frequency of Non-N Detection by the USP-Pirassununga COVID-19 Task Force

The USP-Pirassununga COVID-19 Task Force performed over 130,000 real-time RT-PCR tests for COVID-19 detection for more than 40 different cities from the southeast part of Sao Paulo State so far (31 July 2021). Most of these tests (90,045) were performed with the GeneFinder COVID-19 Plus RealAmp Kit (Osang Healthcare Co., Korea) which uses three different primers sets for viral detection (*N*, *E*, and *RDRP* gene fragments). The Ct values for the three viral genes present similarly in positive samples with high and medium viral load (Cts ranging from 12 to 30). Low viral load samples (Cts > 35) were detected with two (generally *E* and *N*) or only one probe (generally *N* probe).

We noted a specific pattern of *N*^−^, *E*^+^, *RDRP*^+^ in the samples from February 2021 in our diagnostic service, being the first sample detected on 26 February 2021. So far, 69 samples were positive with this pattern from a total of 86,393 tests performed with GeneFinder since April 2020 (until 15 July 2021) by our service. This calls our attention for further investigation because the *N* primer set is the most sensitive probe in our conditions and losing this signal could infer false-negative results especially in low viral load samples.

### 3.2. Genome Sequencing and Identification of N Gene Mutations

Therefore, we sequenced the SARS-CoV-2 genome from 17 representative positive samples, and all were classified as P.1 (or P.1.1) variant. Three different mutations in gene *N* were observed: Del28877-28894 (14/17) causing a deletion of six AA, a substitution of GGG to AAC in 28881-28883 (2/17) changing two AAs and a frameshift mutation caused by a deletion of 28877-28878 (1/17) in Table 1.

The mutations were further characterized in silico for potential effects on protein function. The mutations causing the six AA deletion and two AA substitutions occurred in the Linker region of the nucleocapsid protein.

### 3.3. Data from Other Laboratories from the Sao Paulo State COVID-19 Diagnostic Network

For further validation of these findings, we gathered data from two other centers for COVID-19 diagnostic belonging to the Sao Paulo State Network coordinated by Butantan Institute both using the Osang’s GeneFinder kit. The service from the School of Medicine in Ribeirão Preto from the University of Sao Paulo processed 76,516 samples from January 1st to 30 June 2021, with 31,899 positive cases. From these, 24 samples fitted the criteria of *N*^−^, *E*^+^, *RDRP*^+^ and four of them were sequenced confirming the presence of DEL 28877–28894. Interestingly, the first sample from this lab was first detected on 17 February 2021, almost simultaneously with our first detection. Data from the major laboratory from the Sao Paulo State COVID-19 Diagnostic Network situated in the Butantan Institute processed 389,273 samples with 201,987 positive diagnostics from January 1st to 31 May 2021. From these, 893 samples (0.23% from all cases) presented the pattern of *N*^−^, *E*^+^, *RDRP*^+^ detection by RT-PCR.

### 3.4. Presence and Frequency of N Gene Mutations in the Databank of SARS-CoV2 Sequenced Samples from the Sao Paulo COVID-19 Variant Alert Network

**Figure 1:**
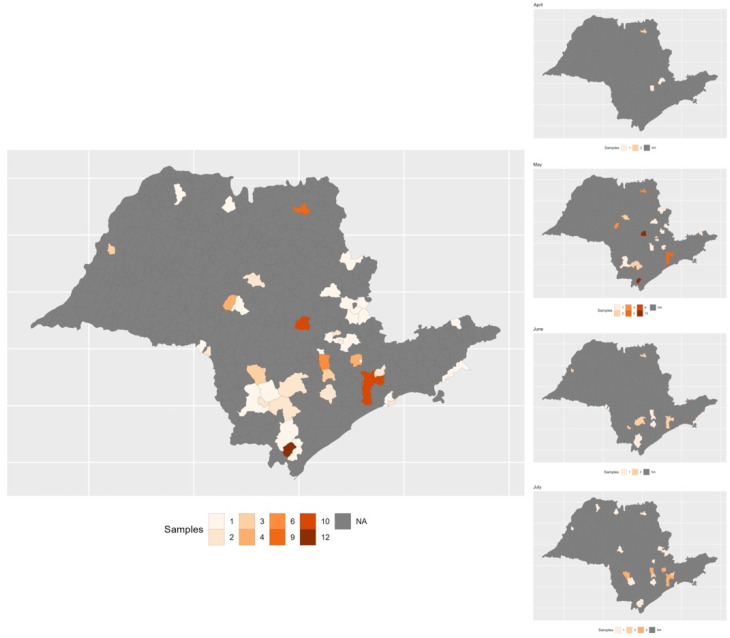
Geolocation of the 111 samples with mutations in the same region of *N* gene and monthly based frequency from April to July 2021.

The Sao Paulo COVID-19 Variant Alert Network sequenced 14,316 SARS-CoV-2 samples (including 1046 sequenced in our lab) and a total of 111 samples (including our 17 already mentioned samples) (Table S1) have mutations in the same region of the *N* gene. The most frequent was DEL 28877–28894 (99 samples) but two other mutations were also found: DEL 28877–28886 (11 samples) and DEL 28847–28886 (1 sample). The mutated SARS-CoV-2 viruses were detected in different cities through the Sao Paulo state (Figure 1).

### 3.5. Characterization of DEL 28877–28894 Mutation in SARS-CoV2

**Figure 2:**
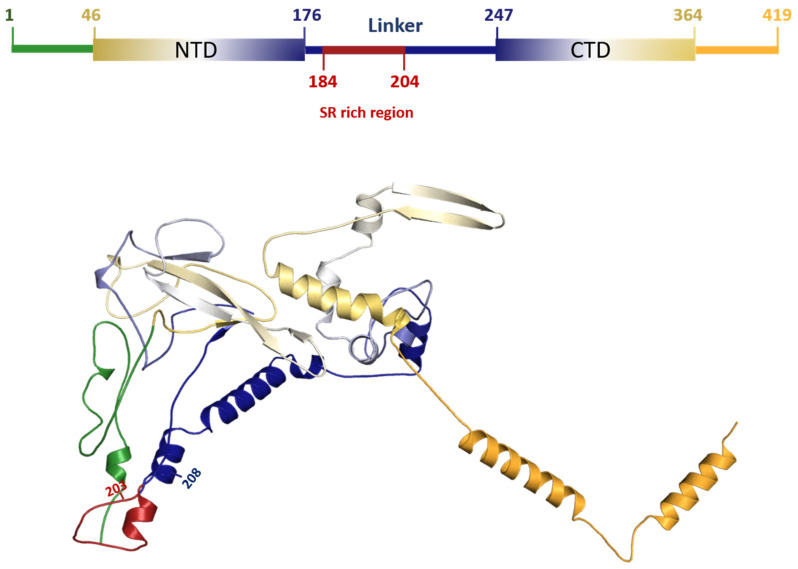
Representative model of protein N indicating the region of the 203–208 deletion, generated by the Robetta server and using the software PyMOL for image generation. The generated model is in compliance with previous work suggesting that the NTD and CTD minimally interact to each other, if at all. They are separated by the linker that is partially extended and with disordered regions have transient α-helices [6,7,8,9]. Deletion of the 203–208 fragment will alter physico-chemical properties that might impact on protein mobility between N- and C-terminal domains, as well as in the RNA-mediated phase separation process.

The DEL 28877–28894 at protein level produced a deletion of six AAs located in the central intrinsic disorder region (IDR) (182–247) that links the NTD (N-terminal domains) to CTD (C-terminal domains). This is the same region where the highest frequency of N protein mutations is reported [2,3,4], including substitutions at amino acids 203 and 204 (also found in this work), which are part of a serine and arginine-rich region comprising residues 184–204 [2,3,4,5]. (Figure 2)

## 4. Discussion

Commercial SARS-CoV-2 real-time RT-PCR kits generally do not disclose the position of primers and probes for the detection sites but here we describe a set of mutations in *N* gene that affect the detection of the *N* gene by the GeneFinder COVID-19 Plus RealAmp Kit used for the diagnostic in Sao Paulo State since the beginning of the COVID-19 pandemics. Although the failure to detect *N* gene, the GeneFinder assay positively call samples by *E* and/or *RdRP* targets, emphasizing the importance of more than one target in a diagnostic RT-PCR kit. The frequency of these *N* mutations affecting GeneFinder *N* gene detection in the epidemiological data from the Sao Paulo state Network for Pandemic Alert of Emerging SARS-CoV-2 variants is low (~0.78%, 111 samples in 14,316) but these are spread all over the state as showed.

Considering that the *N* gene is the most sensitive probe in our conditions, it is plausible to ponder that false-negative results especially in samples with low viral load can happen due to these *N* mutations. In fact, a similar problem was recently demonstrated by Hasan and colleagues using another FDA (Food and Drug Administration) approved SARS-CoV-2 test (Cepheid Xpert Xpress SARS-CoV-2) that uses two viral targets, *N* and *E* genes [10]. In their work, at the end of October 2020, a mutation in the SARS-CoV-2 *N* gene was suspected when Xpert failed to amplify the *N* gene target in a specimen, despite giving a strong positive result (Ct = 19.8) for the *E* gene. They also detected three more samples in the next two months and after sequencing they found a point mutation (C29200A) in these samples. An analogous issue was showed by Artesi and colleagues that found a recurrent mutation at position 26,340 associated with failure of the *E* gene detection by the cobas SARS-CoV-2 test (Roche) [11]. Here, we consistently showed five different *N* gene mutations that affect the detection of *N* gene by the GeneFinder Kit in more than 100 sequenced samples being the DEL 28877–28894 in Gamma variant the most frequent. Our results agree with the data showing the *N* gene as one of the most non-conservative genes in the SARS-CoV-2 genome [12].

The main roles of the multifunctional nucleocapsid (N) protein in SARS-CoV-2 include viral genome packaging and virion assembly, viral transcription, regulation of transcription in infected cells and in suppression of the host innate immune response [2,3,13,14]. The N protein has 419 amino acids, divided into two main domains (N-terminal (NTD) and C-terminal (CTD)), with well-known structures [2,4,5] (Figure 2). The NTD RNA-binding and CTD dimerization domains range from amino acids 46–176 and 247–364, respectively. These regions are interspersed with three other domains that are intrinsically disordered [2,4,5,15]. The 203–208 deletion found in protein N was previously identified in Australia and Malaysia [2], suggesting that it may confer some adaptive advantage to SARS-CoV-2. This advantage can be a consequence of alterations that occur at both protein and RNA levels. The linker region was experimentally predicted as fundamental for RNA-mediated phase separation [16]. In fact, this IDR may be directly involved in protein–protein interactions to promote phase separation with RNA. Additionally, the region rich in serine and arginine at this IDR was predicted to model the physical properties of the resulting condensate [3,17]. At the genomic RNA level, it was reported that RNA-sequence distinct regions of the viral RNA genome can promote either phase separation or solubilization [18]. Interestingly, the N protein-coding region is predicted to be a phase separation promoter [18]. In addition, at RNA level, the 203–208 deletion can also directly impact the mRNA structure by changing its functional half-life and affecting the regulation of protein expression [19]. Altogether, it is feasible to suggest that 203–208 deletion may direct impact on the process of phase separation, leading to possible optimization in the process of packaging and replication.

## 5. Conclusions

Here we demonstrated the existence of mutations in the *N* gene that might affect the use of SARS-CoV2 real-time RT-PCR diagnostic kits impacting the false-negative results. These results provide further evidence that existing variants of SARS-CoV2 might escape molecular detection based on nucleic acid amplification tests, especially those ones using a single target of the virus.

## Figures and Tables

**Table 1:** Mutations in gene N detected by sequencing.

Mutation	Alteration	Frequency
DEL 28877-28894	Deletion	
	N:S202- N:R203- N:G204- N:T205- N:S206-	82% (14/17)
	N:P207-	
G28881A G28882A G28883C	Substitution N:R203K N:G204R	12% (2/17)
DEL 28877-28878	Deletion	
	Frameshift	6% (1/17)

## Data Availability

The data presented in this study are available on request from the corresponding author.

## Supplementary Materials

The following are available online at https://www.mdpi.com/1999-4915/13/12/2474/s1; Table S1: Mutations in gene N detected by sequencing.
