# Correction: Lesbon et al. Nucleocapsid (N) Gene Mutations of SARS-CoV-2 Can Affect Real-Time RT-PCR Diagnostic and Impact False-Negative Results. *Viruses* 2021, *13*, 2474

**DOI:** 10.3390/v14091967

**Published:** 2022-09-05

**Authors:** Jéssika Cristina Chagas Lesbon, Mirele Daiana Poleti, Elisângela Chicaroni de Mattos Oliveira, José Salvatore Leister Patané, Luan Gaspar Clemente, Vincent Louis Viala, Gabriela Ribeiro, Marta Giovanetti, Luiz Carlos Junior de Alcantara, Olivia Teixeira, Maria Cristina Nonato, Loyze Paola Oliveira de Lima, Antonio Jorge Martins, Claudia Renata dos Santos Barros, Elaine Cristina Marqueze, Jardelina de Souza Todão Bernardino, Debora Botequio Moretti, Ricardo Augusto Brassaloti, Raquel de Lello Rocha Campos Cassano, Pilar Drummond Sampaio Correa Mariani, Svetoslav Nanev Slavov, Rafael Bezerra dos Santos, Evandra Strazza Rodrigues, Elaine Vieira Santos, Josiane Serrano Borges, Debora Glenda Lima de La Roque, Joao Paulo Kitajima, Bibiana Santos, Patricia Akemi Assato, Felipe Allan da Silva da Costa, Cecilia Artico Banho, Livia Sacchetto, Marilia Mazzi Moraes, Melissa Palmieri, Fabiana Erica Vilanova da Silva, Rejane Maria Tommasini Grotto, Jayme A. Souza-Neto, Mauricio Lacerda Nogueira, Luiz Lehman Coutinho, Rodrigo Tocantins Calado, Raul Machado Neto, Dimas Tadeu Covas, Simone Kashima, Maria Carolina Elias, Sandra Coccuzzo Sampaio, Heidge Fukumasu

**Affiliations:** 1 Laboratory of Comparative and Translational Oncology, Department of Veterinary Medicine, School of Animal Science and Food Engineering, University of Sao Paulo, Pirassununga 13635-900, São Paulo, Brazil; 2 Butantan Institute, São Paulo 05503-000, São Paulo, Brazil; 3 Functional Genomic Center, Department of Animal Science, Luiz de Queiroz School of Agriculture, University of Sao Paulo, Piracicaba 13418-900, São Paulo, Brazil; 4 Fundação Oswaldo Cruz, FIOCRUZ, Manguinhos 21040-900, Rio de Janeiro, Brazil; 5 Institute of Biological Sciences, Federal University of Minas Gerais, Belo Horizonte 31270-901, Minas Gerais, Brazil; 6 Ribeirao Preto Protein Crystallography Laboratory, School of Pharmaceutical Sciences, University of São Paulo, Ribeirao Preto 14040-903, São Paulo, Brazil; 7 NGS Soluções Genômicas, Piracicaba 13416-030, São Paulo, Brazil; 8 Blood Center of Ribeirão Preto, Ribeirão Preto Medical School, University of São Paulo, Ribeirão Preto 14051-060, São Paulo, Brazil; 9 Mendelics Genomic Analysis, São Paulo 02511-000, São Paulo, Brazil; 10 School of Agricultural Sciences, São Paulo State University, Botucatu 18618-970, São Paulo, Brazil; 11 Laboratório de Pesquisas em Virologia, Departamento de Doenças Dermatológicas, Infecciosas e Parasitárias, Faculdade de Medicina de São José do Rio Preto, São José do Rio Preto 15090-000, São Paulo, Brazil; 12 Coordenação de Vigilância em Saúde—Secretaria Municipal da Saúde, São Paulo 01223-906, São Paulo, Brazil; 13 Laboratory Assistance, Coordination of Primary Care, Municipal Health Department, São Paulo 01223-906, São Paulo, Brazil

## Addition of Two Authors

The authors hereby request the inclusion of two authors (Olivia Teixeira and Maria Cristina Nonato) in the recently published article in *Viruses* entitled “Nucleocapsid (N) gene mutations of SARS-CoV-2 can affect real-time RT-PCR diagnostic and impact false-negative results” [1]. 

As already demonstrated to the Journal, the authors Olivia Teixeira and Maria Cristina Nonato were very important contributors of this article as they were solely responsible for the analysis of the protein structure and the effects of the SARS-CoV-2 mutations on its function at RNA and protein levels. They also collaborated in the discussion of results and the preparation of Figure 2 of the protein structure. Therefore, Olivia Teixeira and Maria Cristina Nonato have to be part of this article as authors. Their names are added between Luiz Carlos Junior de Alcantara and Loyze Paola Oliveira de Lima. All authors have agreed on this change. 

## Additional Affiliations

An additional affiliation, 6, is added in regard to the addition of two authors (Olivia Teixeira and Maria Cristina Nonato). The numbers of other affiliations have been adjusted accordingly. 

Newly added affiliation 6: Ribeirao Preto Protein Crystallography Laboratory, School of Pharmaceutical Sciences, University of São Paulo, Ribeirao Preto 14040-903, São Paulo, Brazil.

In the published publication, there was an error regarding the affiliation for “Dimas Tadeu Covas”. In addition to affiliation “2”, the updated affiliation should include: “8, Blood Center of Ribeirão Preto, Ribeirão Preto Medical School, University of São Paulo, Ribeirão Preto 14051-060, São Paulo, Brazil”.

## Insert New Author Contributions Statement

The contributions of Olivia Teixeira and Maria Cristina Nonato were not included in the original publication. The corrected Author Contributions Statement appears here. 

**Author Contributions:** Conceptualization and designed the experiments: H.F.; performed the analysis: J.C.C.L., M.D.P., O.T. and M.C.N.; analyzed the data: J.C.C.L., H.F., E.C.d.M.O., G.R., L.G.C., O.T. and M.C.N.; writing—original draft preparation: J.C.C.L.; writing—review and editing: J.C.C.L., M.D.P., M.G., S.K. and H.F.; Molecular screening and produced SARS-CoV-2 genomic data: J.C.C.L., M.D.P., E.C.d.M.O., J.S.L.P., L.G.C., V.L.V., M.G., L.C.J.d.A., L.P.O.d.L., A.J.M., C.R.d.S.B., E.C.M., J.d.S.T.B., D.B.M., R.A.B., R.d.L.R.C.C., P.D.S.C.M., S.N.S., R.B.d.S., E.S.R., E.V.S., J.S.B., D.G.L.d.L.R., J.P.K., B.S., P.A.A., F.A.d.S.d.C., C.A.B., L.S., M.M.M., M.P., F.E.V.d.S., R.M.T.G., J.A.S.-N., M.L.N., L.L.C., R.T.C., R.M.N., D.T.C., S.C.S., M.C.E., S.K., H.F. All authors have read and agreed to the published version of the manuscript.

The authors apologize for any inconvenience caused and state that the scientific conclusions are unaffected. This correction was approved by the Academic Editor. The original publication has also been updated.
